# Comparative Analysis of Lenvatinib and Hepatic Arterial Infusion Chemotherapy in Unresectable Hepatocellular Carcinoma: A Multi-Center, Propensity Score Study

**DOI:** 10.3390/jcm10184045

**Published:** 2021-09-07

**Authors:** Jaejun Lee, Ji-Won Han, Pil-Soo Sung, Soon-Kyu Lee, Hyun Yang, Hee-Chul Nam, Sun-Hong Yoo, Hae-Lim Lee, Hee-Yeon Kim, Sung-Won Lee, Jung-Hyun Kwon, Jeong-Won Jang, Chang-Wook Kim, Soon-Woo Nam, Jung-Suk Oh, Ho-Jong Chun, Si-Hyun Bae, Jong-Young Choi, Seung-Kew Yoon

**Affiliations:** 1 The Catholic University Liver Research Center, College of Medicine, The Catholic University of Korea, Seoul 06591, Korea; pwln0516@gmail.com (J.L.); tmznjf@catholic.ac.kr (J.-W.H.); blackiqq@gmail.com (S.-K.L.); oneggu@naver.com (H.Y.); hcnam128@catholic.ac.kr (H.-C.N.); calla0108@naver.com (S.-H.Y.); atom069@naver.com (H.-L.L.); hee82@catholic.ac.kr (H.-Y.K.); zambrotta@catholic.ac.kr (S.-W.L.); doctorkwon@catholic.ac.kr (J.-H.K.); garden@catholic.ac.kr (J.-W.J.); cwkim@catholic.ac.kr (C.-W.K.); drswnam@hanmail.net (S.-W.N.); baesh@catholic.ac.kr (S.-H.B.); jychoi@catholic.ac.kr (J.-Y.C.); yoonsk@catholic.ac.kr (S.-K.Y.); 2 Division of Gastroenterology and Hepatology, Department of Internal Medicine, College of Medicine, Eunpyeong St. Mary’s Hospital, The Catholic University of Korea, Seoul 03382, Korea; 3 Division of Gastroenterology and Hepatology, Department of Internal Medicine, College of Medicine, Seoul St. Mary’s Hospital, The Catholic University of Korea, Seoul 06591, Korea; 4 Division of Gastroenterology and Hepatology, Department of Internal Medicine, College of Medicine, Uijeongbu St. Mary’s Hospital, The Catholic University of Korea, Seoul 11765, Korea; 5 Division of Gastroenterology and Hepatology, Department of Internal Medicine, College of Medicine, Incheon St. Mary’s Hospital, The Catholic University of Korea, Seoul 22711, Korea; 6 Division of Gastroenterology and Hepatology, Department of Internal Medicine, College of Medicine, Bucheon St. Mary’s Hospital, The Catholic University of Korea, Seoul 14647, Korea; 7 Department of Radiology, College of Medicine, Seoul St. Mary’s Hospital, The Catholic University of Korea, Seoul 06591, Korea; oj-cumc@hanmail.net (J.-S.O.); chunray@catholic.ac.kr (H.-J.C.)

**Keywords:** hepatocellular carcinoma, lenvatinib, hepatic arterial infusion chemotherapy, propensity score matching

## Abstract

The comparative efficacy and safety between lenvatinib and hepatic artery infusion chemotherapy (HAIC) in patients with unresectable hepatocellular carcinoma (HCC) is still unclear. This multicenter historical cohort study enrolled 244 patients who were treated with HAIC (*n* = 173) or lenvatinib (*n* = 71) between 2012 and 2020. Propensity score matching (PSM) was performed, and 52 patients were selected per group. Clinical outcomes and safety were compared. Objective response rate (ORR) was not different between the two groups (26.0% vs. 23.1%, *p* = 0.736). Before PSM, the HAIC group had a higher proportion of Child-Pugh B and portal vein tumor, whereas the lenvatinib group had more patients with extrahepatic metastases, which was adjusted after PSM. There were no differences in progression-free survival (PFS) and overall survival (OS) after PSM (HAIC vs. lenvatinib, median PFS, 3.6 vs. 4.0 months, *p* = 0.706; median OS 10.8 vs. 7.9 months, *p* = 0.106). Multivariate Cox-regression showed that alpha-fetoprotein ≤1000 ng/mL was only an associated factor for OS after PSM in all patients (hazard ratio = 0.421, *p* = 0.011). Subgroup analysis for patients with a high tumor burden beyond the REFLECT eligibility criteria revealed that the HAIC group (*n* = 29) had a significantly longer OS than did the lenvatinib group (*n* = 30) (10.0 vs. 5.4 months, *p* = 0.004). More patients in the HAIC group achieved better liver function than those in the lenvatinib group at the time of best responses. There was no difference in the incidence of grade 3 and 4 adverse events between the two groups. Therefore, lenvatinib is comparable to HAIC in terms of ORR and OS in unresectable HCC meeting REFLECT eligibility criteria.

## 1. Introduction

Hepatocellular carcinoma (HCC) is one of the most common causes of cancer-related deaths worldwide [1]. According to the Barcelona Clinic Liver Cancer (BCLC) staging system, HCC can be classified into five stages, and stage C represents unresectable tumors with macrovascular invasion or extrahepatic spread [1]. For patients with BCLC stage C or with BCLC-B who are not suitable for local treatments, systemic therapies have been recommended as the first-line therapy [2].

A recent randomized phase 3 trial showed that lenvatinib, a recently introduced tyrosine kinase inhibitor (TKI), is non-inferior to sorafenib in terms of overall survival (OS) in treatment-naïve unresectable HCC (the REFLECT trial) [3]. In addition, our group recently demonstrated that lenvatinib showed better progression-free survival (PFS) than sorafenib as a salvage treatment after transarterial treatment failure [4], which may be due to differences in molecular targets, including fibroblast growth factor pathways [5]. Nevertheless, the eligibility criteria for tumor burden in the REFLECT trial were only applicable to the selected patients (tumor extent <50% of liver volume, absence of main portal vein tumor thrombosis (PVTT), absence of bile duct invasion) [6], and lenvatinib treatment in patients beyond these criteria showed varying outcomes, dependent on multiple factors, such as previous treatment history or PVTT [7].

Hepatic arterial infusion chemotherapy (HAIC) is considered a treatment option, instead of systemic chemotherapy, to reduce intrahepatic tumor burden in BCLC stage B or C with predominant intrahepatic disease by administering cytotoxic chemotherapeutic agents with high intrahepatic concentrations [8,9]. In addition, it can also be used in the poor responders of transarterial chemoembolization [10,11]. Therefore, HAIC is recommended as a therapeutic option for advanced HCC with vascular invasion in the Japanese guidelines [12]. Previous reports have shown that the control of intrahepatic tumors by HAIC provides survival benefits, even in patients with extrahepatic metastases or Vp 3/4 PVTT [13,14]. Until now, there have been several studies comparing the efficacy of HAIC and sorafenib. A randomized phase 3 trial (SILIUS trial), which compared sorafenib alone to sorafenib plus HAIC, failed to show the superiority of the combination in advanced HCC [15]. However, more recently, another phase 3 randomized trial showed that oxaliplatin-based HAIC plus sorafenib exhibited a survival benefit compared to sorafenib alone in patients with HCC with portal vein invasion [16]. This implies that HAIC may be an effective treatment option in selected patient groups. Moreover, small prospective cohort studies showed that HAIC had a survival benefit compared with sorafenib in patients with advanced HCC with macrovascular invasion (MVI) without extrahepatic metastases [17,18]. Furthermore, a recent large-scale retrospective study using propensity score matching (PSM) analysis also reported that HAIC was superior to sorafenib in patients with advanced HCC with MVI without extrahepatic metastases in terms of OS [19]. However, no previous report has compared the real-world efficacy and safety between lenvatinib and HAIC in unresectable HCC.

Here, we performed a multicenter, historical cohort study in which HAIC and lenvatinib were compared in patients with unresectable HCC in terms of efficacy and safety. We used PSM to correct the various clinical parameters of patients with HCC, including tumor factors. We also analyzed the differences in survival outcomes between lenvatinib and HAIC in subgroup analysis with patients within or beyond the REFLECT eligibility criteria.

## 2. Patients and Methods

### 2.1. Study Population

This study was approved by the Institutional Review Board of the Catholic University of Korea (approval number: XC21RIDI0008), and was performed in accordance with the Declaration of Helsinki. We retrospectively evaluated 244 consecutive patients with unresectable HCC who were treated with HAIC or lenvatinib at five affiliated hospitals in Korea. Patients treated with HAIC were enrolled from November 2012 to November 2020, whereas patients treated with lenvatinib were enrolled from January 2019 to November 2020. HCC was diagnosed by histological or radiological examinations via contrast-enhanced computed tomography (CT) and/or magnetic resonance imaging (MRI). The inclusion criteria were as follows: (1) confirmed intermediate to advanced HCC, but ineligible for surgical resection; (2) age ≥18 years; and (3) Eastern Cooperative Eligibility criteria (ECOG) performance status score of ≤2. The exclusion criteria were as follows: (1) lack of follow-up visits after the start of the treatment; (2) a treatment duration of <2 weeks for lenvatinib-treated patients; (3) less than two cycles of HAIC treatment for HAIC-treated patients; and (4) history of malignancy other than HCC in the previous 5 years.

### 2.2. Treatment Protocol

Lenvatinib was administered once daily at a dose of 8 mg for patients weighing <60 kg, and at a dose of 12 mg for patients weighing >60 kg. HAIC was performed as previously described [18,20]. The chemotherapy regimen consisted of 5-fluorouracil (5-FU) at a dose of 500 mg/m^2^/day, and cisplatin at a dose of 60 mg/m^2^/day. 5-FU was administered for 5 h daily on days 1–3 and cisplatin for 2 h on day 1 or 2. For arterial chemo-infusion, the catheter was inserted through the femoral artery and its tip was advanced to the common or proper hepatic artery; the other end of the tip was connected to the chemoport implanted in the subcutaneous pocket of the inguinal region. Each session was delivered every 3–4 weeks via an implantable port system.

### 2.3. Response Evaluation

We classified the patients according to the BCLC stage, which was based on the radiologic and laboratory findings at the time of study enrollment. Imaging studies (CT or MRI) were conducted every 4–12 weeks for lenvatinib treatment and every 2–3 cycles of HAIC treatment for response evaluation. The assessment was conducted according to the modified Response Evaluation Criteria in Solid Tumors (mRECIST) as in our previous study [21]. OS was calculated from the start of drug administration until death or the last follow-up day. PFS was calculated as the time from the start of drug administration until disease progression, or drug cessation due to any cause in the absence of disease progression. The objective response rate (ORR) was calculated as the sum of the “complete response” and “partial response” at the response evaluation. The disease control rate (DCR) was calculated as the sum of the complete response (CR), partial response (PR), and stable disease (SD). The treatment response was defined as the best response during treatment. We categorized tumor types into nodular, massive, and diffuse types according to Eggel’s classification [22]. Thereafter, massive and diffuse types were classified as non-nodular types. The modified albumin-bilirubin (mALBI) score was also measured to assess residual liver function at the end of each treatment as previously described [23], and with the following formula: mALBI = (log_10_ serum total bilirubin [µmol/L] × 0.66) + (serum albumin [g/L]–0.085). Patients were divided into four groups according to the mALBI score: grade 1 (<−2.60), grade 2a (2.60 ALBI score <−2.27), grade 2b (−2.27), and grade 3 (<−1.39). Adverse events were assessed according to the Common Terminology Criteria for Adverse Events version 4.0 [24].

### 2.4. Propensity-Score Matching (PSM)

We used PSM to adjust for differences in baseline characteristics between the HAIC (*n* = 173) and lenvatinib (*n* = 71) groups. Variables known to be related to the prognosis of HCC were selected for PSM, and included the ECOG, age, Child-Pugh score, extrahepatic metastasis and vascular invasion, intrahepatic tumor size, tumor type (nodular and non-nodular), and BCLC stage.

One-to-one nearest-neighbor matching within a caliper size of 0.20 was used. PSM analyses resulted in the selection of 52 patients in each group.

### 2.5. Statistical Analysis

All statistical analyses were performed using R statistical software (version 4.0.3; R Foundation Inc., Vienna, Austria; http://cran.r-project.org (access on 3 September 2021)) and SPSS version 23.0 software (SPSS, Chicago, IL, USA). The median clinical parameter values were calculated and the interquartile ranges were documented. The student’s t-test was used to compare continuous variables between the two groups. The Kaplan–Meier method was used for survival analyses, including OS and PFS, and differences were examined using the log-rank test. Cox regression analyses were performed to identify the factors associated with survival outcomes, and factors with *p* < 0.01 in univariate analysis were included in multivariate analysis. The therapeutic efficacy was demonstrated by the ORR and DCR, which were compared using the chi-square test. Statistical significance was defined as *p*-values < 0.05.

## 3. Results

### 3.1. Baseline Characteristics

The baseline characteristics are presented in Table 1. Among the 244 patients, 173 received HAIC and 71 received lenvatinib. The patients with HAIC were younger than those with lenvatinib (mean, 58.3 vs. 63.1 years old; *p* = 0.001). In addition, the percentage of Child-Pugh B was higher in the HAIC group than in the lenvatinib group (52.6% vs. 25.4%, *p* < 0.001). The etiology was also significantly different between the two groups (*p* = 0.019); the HAIC group had a higher percentage of hepatitis B virus etiology (77.5% vs. 59.2%) and a lower percentage of alcohol etiology (9.2% vs. 22.5%). The median alpha-fetoprotein (AFP) level was higher (976 vs. 662.2 ng/mL, *p* = 0.037) in the HAIC group. The maximum tumor size was larger in the HAIC group (mean, 9.7 vs. 7.7 cm; *p* = 0.005), and the non-nodular type was more frequent in the HAIC group (63.0% vs. 28.2%, *p* < 0.001), as was PVTT (75.7% vs. 46.5%, *p* < 0.001). In contrast, extrahepatic metastasis was more frequent in the lenvatinib group (52.1% vs. 27.2%, *p* < 0.001). A history of previous HCC treatment was more common in the lenvatinib group than in the HAIC group (83.1% vs. 51.4%, *p* < 0.001). Among those with a history of previous HCC treatment, 6 patients in the lenvatinib group and 12 patients in the HAIC group received either TKI or immune checkpoint inhibitor previously (8.5% vs. 6.9%, *p* = 0.681). Detailed previous treatment histories are presented in Table S1.

PSM was performed to adjust these differences in baseline characteristics between the two groups (Table 1), and 104 patients were selected for analysis after PSM (52 patients per group). No significant differences were observed between the two groups after PSM, except for median PIVKA-II levels (*p* = 0.046).

### 3.2. Treatment Responses

When we assessed treatment responses using the best response during treatment in the PSM cohort, 4 (7.7%) patients in the HAIC group and 2 (3.8%) patients in the lenvatinib group achieved CR, and 11 (21.2%) patients in the HAIC group and 10 (19.2%) patients in the lenvatinib group achieved PR. There was no statistical difference in ORR between the two groups (HAIC, 28.8% vs. lenvatinib, 23.1%; *p* = 0.502) (Table 2), although the DCR was different between the two groups (73.1% in the HAIC group and 51.9% in the lenvatinib group; *p* = 0.026), and especially the proportion of stable disease was higher in the HAIC group. These tendencies were also observed in the entire cohort without PSM (Table 2), which might be due to the high accumulation of chemotherapeutics within the tumor in HAIC.

To assess early biological responses to treatment options, we analyzed the proportion of early AFP responses 4 weeks after the treatment, based on more than 20% or 50% reductions in AFP levels compared with the baseline levels, which are the most studied definitions [25]. As a result, the proportion of ≥20% AFP reduction at 4 weeks of treatment was not different between the lenvatinib and HAIC groups (Figure S1A). Similarly, the proportion of ≥50% AFP reduction at 4 weeks of treatment was also not significantly different between the two groups (Figure S1B). These findings are in line with the results of PFS, suggesting that early biological responses to lenvatinib might also be comparable to those of HAIC.

### 3.3. Survival Outcomes

**Figure 1:**
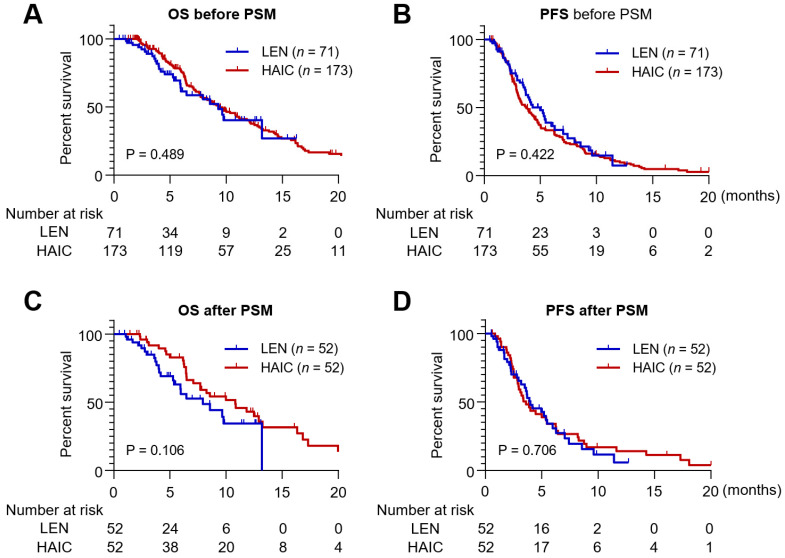
Kaplan–Meier survival curves analyzing OS and PFS in the HAIC and lenvatinib groups before (*n* = 244) and after PSM (*n* = 104). (**A**) Overall survival (OS) of patients treated with lenvatinib and HAIC in the entire cohort. (**B**) Progression free survival (PFS) of patients treated with lenvatinib and HAIC in the entire cohort. (**C**) OS of patients treated with lenvatinib and HAIC following propensity score matching (PSM). (**D**) PFS of patients treated with lenvatinib and HAIC following PSM. PSM: propensity score matching.

We first compared the OS and PFS in the entire cohort without PSM. The median follow-up durations for the HAIC and lenvatinib groups were 6.9 and 4.8 months, respectively (*p* < 0.001), and the median treatment duration for HAIC and lenvatinib was 2.9 and 2.6 months, respectively (*p* = 0.159). The median OS was compared between the two groups, and no statistical difference was observed (HAIC, median of 9.4 months; 95% confidence interval [CI], 7.4–11.4 vs. lenvatinib, median of 9.3 months; 95% CI, 6.8–11.8; *p* = 0.489) (Figure 1A). The median PFS was 3.7 months in the HAIC group (95% CI, 3.0–4.5) and 4.3 months in the lenvatinib group (95% CI, 2.9–5.7), with no statistical significance (*p* = 0.422) (Figure 1B).

After PSM, the median treatment duration did not differ significantly between the two groups (median of 2.9 months in the HAIC group and 2.5 months in the lenvatinib group; *p* = 0.150), either. In contrast, the median follow-up duration was significantly different (median of 7.7 months in the HAIC group and 4.2 months in the lenvatinib group; *p* < 0.001). During the follow-up period, 35 (67.3%) patients in the lenvatinib group and 42 (80.8%) patients in the HAIC group experienced disease progression or death. Although there was a tendency for longer OS in the HAIC group compared to the lenvatinib group (HAIC, median of 10.8 months, 95% CI, 6.9–14.8 vs. lenvatinib, median of 7.9 months, 95% CI, 4.2–11.7), the difference was not statistically significant (*p* = 0.106) (Figure 1C). Moreover, the PFS did not differ significantly between the two groups with median of 4.0 months (95% CI, 2.5–5.5) in the lenvatinib group, and of 3.6 months (95% CI, 2.6–4.6) in the HAIC group (*p* = 0.706) (Figure 1D).

When we compared OS and PFS between lenvatinib and HAIC groups only in patients with non-viral HCC (Figure S2), there was no significant difference, which is consistent with analyses using the entire cohort, suggesting that our results may be applied regardless of etiology, although future studies are needed.

### 3.4. Factors Contributing to Survival Outcomes

Univariate and multivariate analyses were performed using the Cox proportional hazard model to identify factors associated with OS and PFS (Table 3) in the entire cohort. The cut-off value of AFP was determined to be 1000 ng/mL according to the analysis in our previous study [4]. In univariate analyses, age >60 years, intrahepatic maximal tumor size of ≤5 cm, absence of extrahepatic metastasis, AFP levels ≤1000 ng/mL, and Child–Pugh class A were factors associated with favorable OS. In multivariate analyses, extrahepatic metastasis (hazard ratio (HR), 1.6; 95% CI, 1.1–2.3; *p* = 0.014), Child–Pugh class A (HR, 0.7; 95% CI, 0.5–0.9; *p* = 0.028), and AFP levels ≤1000 ng/mL (HR, 0.7; 95% CI, 0.5–0.9; *p* = 0.030) were significant factors associated with OS. Regarding PFS, age ≤ 60 years, intrahepatic maximal tumor size ≤5 cm, extrahepatic metastasis, Child–Pugh class A, and AFP levels ≤1000 ng/mL were significant factors in univariate analyses. In multivariate analyses, Child–Pugh class A (HR, 0.7; 95% CI, 0.5–0.9; *p* = 0.010) and AFP levels ≤1000 ng/mL (HR, 0.7; 95% CI, 0.5–0.9; *p* = 0.026) were significant factors associated with PFS.

Next, we performed subgroup analyses comparing OS between lenvatinib and HAIC groups according to the factors that could be associated with survival outcomes (Figure S3). Most subgroups did not show significant differences in HR between the two groups. However, patients in the lenvatinib group with macrovascular invasion (HR, 1.8; 95% CI, 1.0–3.0; *p* = 0.032), maximal intrahepatic tumor size > 5 cm (HR, 2.0; 95% CI, 1.2–3.2; *p* = 0.008), or AFP level > 1000 ng/mL (HR, 1.8; 95% CI, 1.0–3.1; *p* = 0.034) showed inferior OS outcomes compared to the HAIC group. For PFS, the lenvatinib group showed better PFS than the HAIC group in patients with extrahepatic metastasis (HR, 0.5; 95% CI, 0.3–0.8; *p* = 0.003) (Figure S4).

### 3.5. Patients with Tumor Burden beyond the REFLECT Eligibility Criteria

In the REFLECT trial, the eligibility criteria for tumor burden were strictly selected and comprised the following: Tumor extent < 50% of liver volume, absence of main PVTT, and absence of bile duct invasion. Patients with a tumor burden exceeding the REFLECT eligibility criteria were demonstrated as “REFLECT (−),” and those with a tumor burden within the REFLECT eligibility criteria were demonstrated as “REFLECT (+).” In the entire cohort, the ORR was not significantly different between the REFLECT (+) and REFLECT (−) groups (30.1% vs. 23.0%, *p* = 0.225).

**Figure 2:**
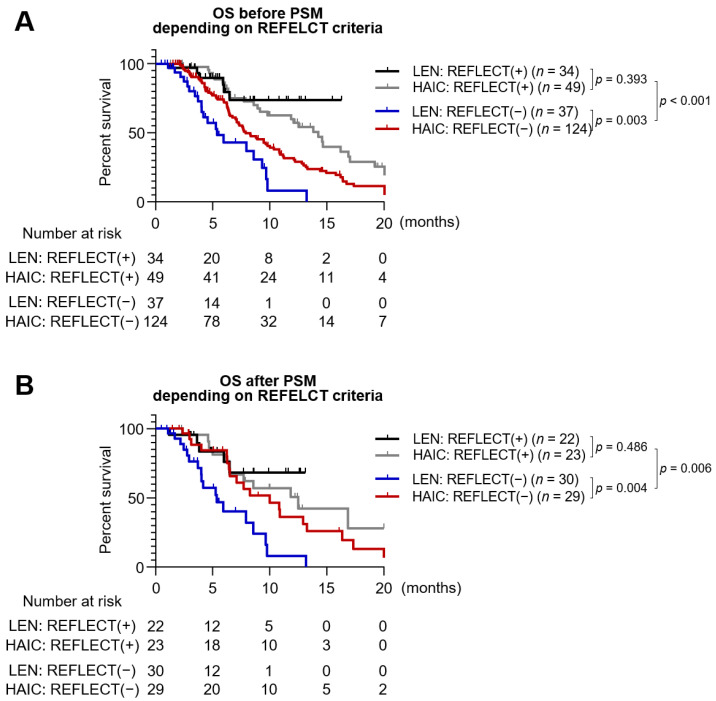
Kaplan–Meier survival curves analyzing OS according to the meeting the RELECT criteria and types of treatment. (**A**) OS in the entire cohort (*n* = 244). (**B**) OS in the PSM cohort (*n* = 104). REFLECT (+) means “meeting the REFLECT criteria”.

Figure 2 shows the Kaplan–Meier survival curve according to the REFLECT eligibility criteria and the type of treatment. In the entire cohort, REFLECT (+) patients showed better outcomes in survival rate compared to REFLECT (−) patients (median of 14.6 vs. 7.7 months, *p* < 0.001) (Figure 2A). Among REFLECT (−) patients, the HAIC group showed significantly higher OS than the lenvatinib group (median of 7.9 vs. 5.4 months, *p* = 0.003). When only considering patients after PSM, REFLECT (+) patients also showed longer OS than REFLECT (−) PSM patients (median of 12.5 vs. 7.7 months, *p* = 0.006). Furthermore, longer OS of the HAIC group compared to the lenvatinib group was also observed among REFLECT (−) PSM patients (median of 10.0 vs. 5.4 months, *p* = 0.004) (Figure 2B).

### 3.6. Treatment-Related Toxicity

Table 4 shows the adverse events, grade ≥ 3 in the lenvatinib and HAIC groups after PSM. Elevation of aspartate aminotransferase was the most common severe adverse event (11/52, 21.2%) in the HAIC group, followed by the elevation of alanine aminotransferase (7/52, 13.5%) and hyperbilirubinemia (7/52, 13.5%). In the lenvatinib group, hypertension (5/52, 9.6%), thrombocytopenia (5/52, 9.6%), diarrhea (5/52, 9.6%) and hepatic encephalopathy (5/52, 9.6%) were the most common severe adverse events. Proteinuria was observed in three patients in the lenvatinib group. Overall, the prevalence of severe adverse events was not significantly different between the two groups, with 48.1% (25/52) in the HAIC group and 44.2% (23/52) in the lenvatinib group (*p* = 0.694).

### 3.7. Liver Function after Lenvatinib or HAIC

The residual liver function was evaluated using the Child-Pugh score and mALBI in each group following PSM (Table 5). Evaluation of residual liver function was done at the point of which the best treatment response was achieved. For those who had not undergone response evaluation, liver function was reviewed at one month after drug administration. As a result, 23 (44.2%) patients in the lenvatinib group, and 35 (67.3%) patients in the HAIC group showed Child-Pugh A liver function at the time of best responses, which was significantly higher in the HAIC group (*p* = 0.018). Furthermore, more patients in the HAIC group achieved better liver function by mALBI ≤ 2a than those in the lenvatinib group (48.1% vs. 25%, respectively, *p* = 0.015) (Table 5). Overall, HAIC tended to preserve hepatic reserve compared to lenvatinib.

Figure S5 shows the survival outcomes between groups with and without subsequent therapy in the PSM cohort. We only considered patients who discontinued the lenvatinib or HAIC therapies (*n* = 93). Forty patients who received lenvatinib or HAIC treatment received subsequent therapy and exhibited a median OS of 10.833 months, which was significantly longer than that of patients without subsequent therapy (*n* = 53; median OS, 6.267 months; *p* = 0.033). The number of patients who underwent subsequent therapy was significantly different between lenvatinib and HAIC groups (*p* = 0.015) (Table S2). Fourteen (30.4%) patients in the lenvatinib group and 26 (55.3%) in the HAIC group underwent subsequent therapy. Nivolumab (*n* = 4) was the most common choice for lenvatinib failure patients, whereas sorafenib (*n* = 11) was the most frequently selected drug for subsequent therapy after HAIC treatment.

## 4. Discussion

To the best of our knowledge, this is the first report to compare the real-world outcomes of lenvatinib and HAIC in patients with unresectable HCC. There was no statistically significant difference in OS and PFS between the lenvatinib and HAIC groups before and after PSM. The REFLECT eligibility criteria included patients with a tumor extent <50% of the liver volume, absence of main PVTT, and absence of bile duct invasion [3]. In patients beyond the REFLECT eligibility criteria, the HAIC group showed better OS than the lenvatinib group before and after PSM. There was no significant difference in severe adverse events between the two groups. These results suggest that lenvatinib and HAIC have similar efficacy and safety in unresectable HCC; however, selected groups with high intrahepatic tumor burden and PVTT may benefit from HAIC.

The REFLECT trial demonstrated that lenvatinib is not inferior to sorafenib as a first-line treatment in unresectable HCC in terms of OS (HR, 0.9; median of 13.6 months vs. 12.3 months) [3]. In this study, the REFLECT (+) group showed a mean OS of 14.6 months in the entire cohort and 12.5 months in the PSM cohort, which is consistent with the result from REFLECT trial. Regarding ORR, the total lenvatinib group in our study showed an ORR of 23.9% (by mRECIST), which is compatible with recent real-world studies [4,26], and lower than that of the REFLECT trial. However, even for REFLECT (+) patients in this study, they showed ORR of 30.7% (by mRECIST), which is lower than that in REFLECT trial. [3] This may be due to the inclusion of a higher number of treatment-experienced patients in our study.

Regarding liver function following the treatment, lenvatinib significantly deteriorated liver function between baseline and week 2, and baseline to week 4, as measured by ALBI grade [27]. Preserved liver function at baseline in sequential treatment following TKI predicted improved prognosis [28], and early decline of liver function was associated with poor prognosis in unresectable HCC [27]. In agreement with this previous study, we observed a tendency for worsening of liver function in the lenvatinib group than in the HAIC group when showing best treatment responses (Child-Pugh A, 44.2% vs. 67.3%; *p* = 0.018, mALBI ≤ 2a, 25% vs. 48.1%; *p* = 0.015), resulting in a lower rate of inclusion following subsequent therapy in the Lenvatinib group (55.3% vs. 30.4%, *p* = 0.015). We also showed that subsequent treatment following lenvatinib or HAIC was associated with longer OS, which emphasizes the importance of residual function following the treatments. Thus, HCC treatment in patients with poor liver function, such as Child-Pugh B in patients with advanced stage, remains a clinically unmet need. Although it is not generally recommended as a first-line treatment, HAIC is recommended as a therapeutic option for advanced HCC with vascular involvement, especially in the Japanese guidelines [12]. The Taiwanese and Korean guidelines suggest HAIC as an option for selected patients [29,30]. TKIs, including sorafenib or lenvatinib, are generally used in patients with Child-Pugh A, whereas HAIC can also be used in Child-Pugh B patients. A previous report suggested that HAIC does not significantly reduce liver function in Child-Pugh A patients [31]. Furthermore, HAIC improved liver function in responders when administered in Child-Pugh B patients, which might be linked to the resolution of vascular invasion [32,33]. Thus, HAIC may have clinical benefits in patients with poor liver function.

Previous studies have investigated the combination or sequential application of TKIs and HAIC. A recent randomized study, the SILIUS trial, compared sorafenib versus sorafenib plus HAIC in patients with unresectable HCC, including extrahepatic metastases [15]. Although there was no significant difference in OS between the groups, subgroup analyses showed that the combination treatment had a survival benefit in patients with main portal vein invasion [15], which is consistent with other previous reports [16,34]. Since lenvatinib seems to have a better tumor response rate than sorafenib, future studies are needed to identify the clinical benefit of the combination of lenvatinib and HAIC, especially in patients with vascular invasion. In this regard, a recent retrospective study showed that the lenvatinib, toriplimab, and HAIC combination regimen is superior to lenvatinib alone in terms of PFS and OS [35]. Furthermore, the survival benefit of sequential therapy, both TKI after HAIC failure or HAIC after TKI failure, remains unclear. A retrospective study showed that sorafenib treatment after HAIC failure had a higher survival rate than HAIC alone [36]. In contrast, HAIC improved survival after sorafenib failure [37], which demonstrated the potential role of sequential application of each treatment. Indeed, a previous study highlighted the effect of targeting intrahepatic lesions in prolonging survival following sorafenib treatment [38], and systemic therapies such as regorafenib and nivolumab showed poor responses rates following sorafenib treatment [39]. Therefore, future studies should investigate this sequential strategy in lenvatinib settings. Of note, continuing treatment following lenvatinib failure provided a survival benefit, suggesting that an effective post-progression treatment following lenvatinib failure still needs to be developed [40].

Despite the retrospective design, our study is the first to show comparable clinical outcomes of lenvatinib and HAIC in patients with unresectable HCC, although HAIC had better survival outcomes in selected patient groups such as patients beyond REFLECT criteria. Future large-scale, prospective studies are needed to validate our results.

## Figures and Tables

**Table 1:** Baseline characteristics of the study population.

Before PSM				After PSM		
Treatment	Lenvatinib (*n* = 71)	HAIC (*n* = 173)	*p*-Value	Lenvatinib (*n* = 52)	HAIC (*n* = 52)	*p*-Value
Male sex	62 (87.3)	150 (86.7)	1.000	47 (90.4)	45 (86.5)	0.760
Age (years)	63.1 ± 11.5	58.3 ± 10.2	0.001	61.0 ± 11.2	61.2 ± 11.6	0.939
Child-Pugh			<0.001			1.000
A	53 (74.6)	82 (47.4)		34 (65.4)	33 (63.5)	
B	18 (25.4)	91 (52.6)		18 (34.6)	19 (36.5)	
Etiology			0.019			0.585
HBV	42 (59.2)	134 (77.5)		32 (61.5)	37 (71.2)	
HCV	7 (9.9)	13 (7.5)		4 (7.7)	5 (9.6)	
Alcohol	16 (22.5)	16 (9.2)		12 (23.1)	8 (15.4)	
Others	6 (8.5)	10 (5.8)		4 (7.7)	2 (3.8)	
AFP (ng/mL)	662.2 (37.5–8000.2)	976 (57.2–13,670)	0.037	1479.3 (66.5–11,987)	308.51 (29–12,979.5)	0.458
PIVKA (mAU/mL)	1648.5 (107.9–20,154.9)	1725 (353–14,845)	0.673	5850.5 (130.8–25,629.3)	872 (405.5–4796.7)	0.046
Albumin (g/dL)	3.7 ± 0.5	3.4 ± 0.5	0.001	3.5 ± 0.5	3.6 ± 0.5	0.527
Platelet (10^9^/L)	168.8 ± 97.7	174.9 ± 104.7	0.678	164.2 ± 100.1	176.0 ± 105.8	0.560
Maximal tumor size (cm)	7.7 ± 5.3	9.7 ± 4.8	0.005	8.0 ± 5.0	8.1 ± 4.8	0.934
Tumor type			<0.001			0.549
Nodular	51 (71.8)	64 (37.0)		33 (63.5)	29 (55.8)	
Non-nodular	20 (28.2)	109 (63.0)		19 (36.5)	23 (44.2)	
PVTT	33 (46.5)	131 (75.7)	<0.001	29 (55.8)	29 (55.8)	1.000
Extrahepatic metastasis	37 (52.1)	47 (27.2)	<0.001	20 (38.5)	24 (46.2)	0.552
BCLC			0.408			0.625
B	14 (19.7)	25 (14.5)		12 (23.1)	9 (17.3)	
C	57 (80.3)	148 (85.5)		40 (76.9)	43 (82.7)	
ECOG			0.160			0.578
0	28 (39.4)	80 (46.2)		19 (36.5)	18 (34.6)	
1	39 (54.9)	74 (42.8)		30 (57.7)	28 (53.8)	
2	4 (5.6)	19 (11.0)		3 (5.8)	6 (11.5)	
Previous treatment history	59 (83.1)	89 (51.4)	<0.001	40 (76.9)	30 (57.7)	0.060

Data are presented as *n* (%), mean ± SD, or median (IQR). PSM: propensity score matching, HAIC: hepatic arterial infusion chemotherapy, HBV: hepatitis B virus, HCV: hepatitis C virus, AFP: alpha-fetoprotein, PIVKA: protein induced by vitamin K antagonist, PVTT: portal vein tumor thrombosis, BCLC: Barcelona Clinic Liver Cancer, ECOG: Eastern Cooperative Oncology Group.

**Table 2:** Treatment responses before and after PSM.

Before PSM				After PSM		
	Lenvatinib (*n* = 71)	HAIC (*n* = 173)	*p*-Value	Lenvatinib (*n* = 52)	HAIC (*n* = 52)	*p*-Value
Treatment responses			0.292			0.583
CR	2 (2.8)	6 (3.5)		2 (3.8)	4 (7.7)	
PR	15 (21.1)	39 (22.5)		10 (19.2)	11 (21.2)	
SD	24 (33.8)	89 (51.4)		15 (28.8)	23 (44.2)	
PD	20 (28.2)	38 (22.0)		16 (30.8)	13 (25.0)	
NA	10 (14.1)	1 (0.6)		9 (17.3)	1 (1.9)	
ORR	17 (23.9)	45 (26.0)	0.736	12 (23.1)	15 (28.8)	0.502
DCR	41 (57.7)	134 (77.5)	0.002	27 (51.9)	38 (73.1)	0.026

Data are presented as *n* (%). PSM: propensity score matching, HAIC: hepatic arterial infusion chemotherapy, CR: complete response, PR: partial response, SD: stable disease, PD: progressive disease, NA: not available, ORR: objective response rate, DCR: disease-controlled rate.

**Table 3:** Univariate and multivariate analyses of the factors influencing OS and PFS in the entire cohort.

Variables	Overall Survival			Progression-Free Survival		
	Univariate (*p*-Value)	Multivariate (*p*-Value)	HR (95% CI)	Univariate (*p*-Value)	Multivariate (*p*-Value)	HR (95% CI)
Lenvatinib vs. HAIC	0.490			0.424		
Age ≤ 60 years	0.005	0.098	1.4 (0.9–1.9)	0.010	0.105	1.3 (0.9–1.7)
HBV vs. non-HBV	0.516			0.227		
Tumor size ≤ 5 cm	0.010	0.257	0.8 (0.5–1.2)	0.026	0.379	0.9 (0.6–1.2)
Macrovascular invasion	0.211			0.108		
Extrahepatic metastasis	0.017	0.014	1.6 (1.1–2.3)	0.042	0.061	1.3 (0.9–1.8)
Child class A	0.002	0.028	0.7 (0.5–0.9)	<0.001	0.010	0.7 (0.5–0.9)
AFP ≤ 1000	0.001	0.030	0.7 (0.5–0.9)	<0.001	0.026	0.7 (0.5–0.9)
PIVKA-II ≤ 1000	0.067	0.353	0.8 (0.6–1.2)	0.137		

HAIC: hepatic arterial infusion chemotherapy, HBV: hepatitis B virus, AFP: alpha-fetoprotein, PIVKA-II: protein induced by vitamin K antagonist-II, HR: hazard ratio, CI: confidence interval.

**Table 4:** Grade ≥3 AEs associated with lenvatinib or HAIC treatment after PSM.

Adverse Event	HAIC (*n* = 52)	Lenvatinib (*n* = 52)	*p*-Value
AE grade ≥3 (overlapped)	25 (48.1)	23 (44.2)	0.694
HFSR	0 (0)	2 (3.8)	
Hypertension	0 (0)	5 (9.6)	
Nephrotoxicity			
Proteinuria	0 (0)	3 (5.8)	
Elevated creatinine	2 (3.8)	0 (0)	
Hematologic			
Anemia	4 (7.7)	1 (1.9)	
Neutropenia	1 (1.9)	0 (0)	
Thrombocytopenia	2 (3.8)	5 (9.6)	
Laboratory			
Hyperbilirubinemia	7 (13.5)	3 (5.8)	
AST	11 (21.2)	2 (3.8)	
ALT	7 (13.5)	1 (1.9)	
Gastrointestinal			
Nausea/vomiting	3 (5.8)	2 (3.8)	
Diarrhea	2 (3.8)	5 (9.6)	
Decreased appetite	3 (5.8)	2 (3.8)	
Hepatic encephalopathy	3 (5.8)	5 (9.6)	
Fatigue	3 (5.8)	2 (3.8)	
Dyspnea	0 (0)	1 (1.9)	
Abdominal pain	1 (1.9)	0 (0)	

The data are presented as *n* (%). AE: adverse event, HAIC: hepatic arterial infusion chemotherapy, HFSR: hand foot skin reaction, AST: aspartate aminotransferase, ALT: alanine transaminase.

**Table 5:** Residual liver function at the time of best treatment response.

	Lenvatinib (*n* = 52)	HAIC (*n* = 52)	*p*
Child-Pugh class A	23 (44.2)	35 (67.3)	0.018
mALBI grade ≤2a	13 (25.0)	25 (48.1)	0.015

The data are presented as *n* (%). Adm, Administration, mALBI, modified albumin-bilirubin.

## Data Availability

Data is contained within the article.

## Supplementary Materials

The following are available online at https://www.mdpi.com/article/10.3390/jcm10184045/s1, Figure S1: Graphs comparing early AFP response (≥20% reduction of AFP at 4 weeks of treatment (A), and ≥50% reduction of AFP at 4 weeks of treatment (B), in each treatment group; Figure S2: Kaplan-Meier curves of non-viral HCC comparing lenvatinib and HAIC before and after PSM; Figure S3: Forest plot comparing lenvatinib and HAIC group which representing hazard ratio, *p*-value, and median OS in each subgroup; Figure S4: Forest plot comparing lenvatinib and HAIC group which representing hazard ratio, *p*-value, and median PFS in each subgroup; Figure S5: Kaplan-Meier curve representing survival difference between patients with salvage therapy and without salvage therapy at the end of HAIC or lenvatinib treatment, in PSM cohort; Table S1: Previous treatment history before lenvatinib or HAIC treatment classified according to the BCLC stage; Table S2: Salvage therapy following lenvatinib or HAIC.
