# Supplementary material for: Comparative Analysis of Lenvatinib and Hepatic Arterial Infusion Chemotherapy in Unresectable Hepatocellular Carcinoma: A Multi-Center, Propensity Score Study
Source: J Clin Med. 2021 Sep 7;10(18):4045. doi: 10.3390/jcm10184045 (PMC8464794; doi:10.3390/jcm10184045)
Supplement: Supplementary file 1 [file jcm-10-04045-s001.zip › jcm-1321395-supplementary.pdf]

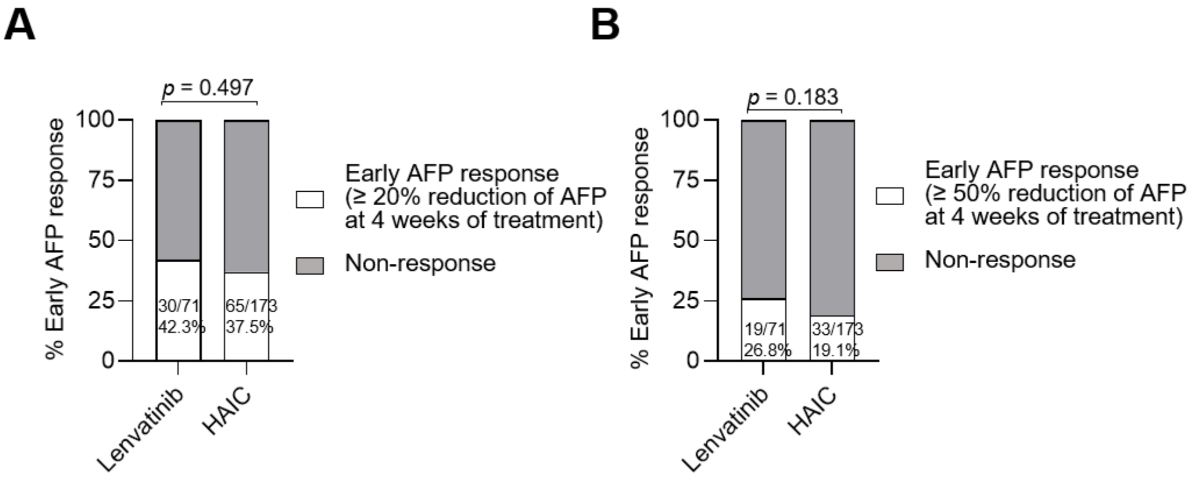

Figure S1. Graphs comparing early AFP response (≧20% reduction of AFP at 4 weeks of treatment (**A**), and ≧50% reduction of AFP at 4 weeks of treatment (**B**), in each treatment group. AFP: alpha-fetoprotein; HAIC: hepatic arterial infusion chemotherapy.

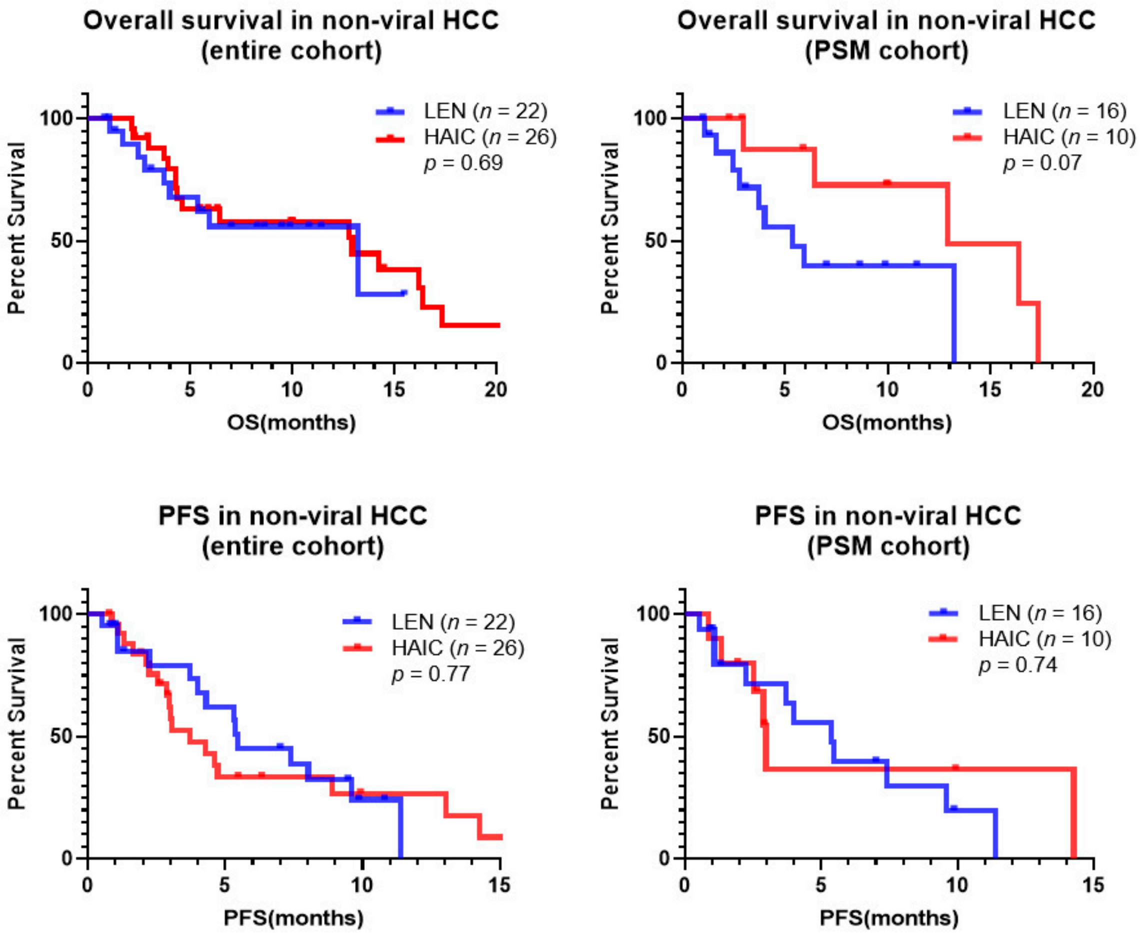

Figure S2. Kaplan-Meier curves of non-viral HCC comparing lenvatinib and HAIC before and after PSM. HCC: hepatocellular carcinoma; PSM: . Propensity score matching.

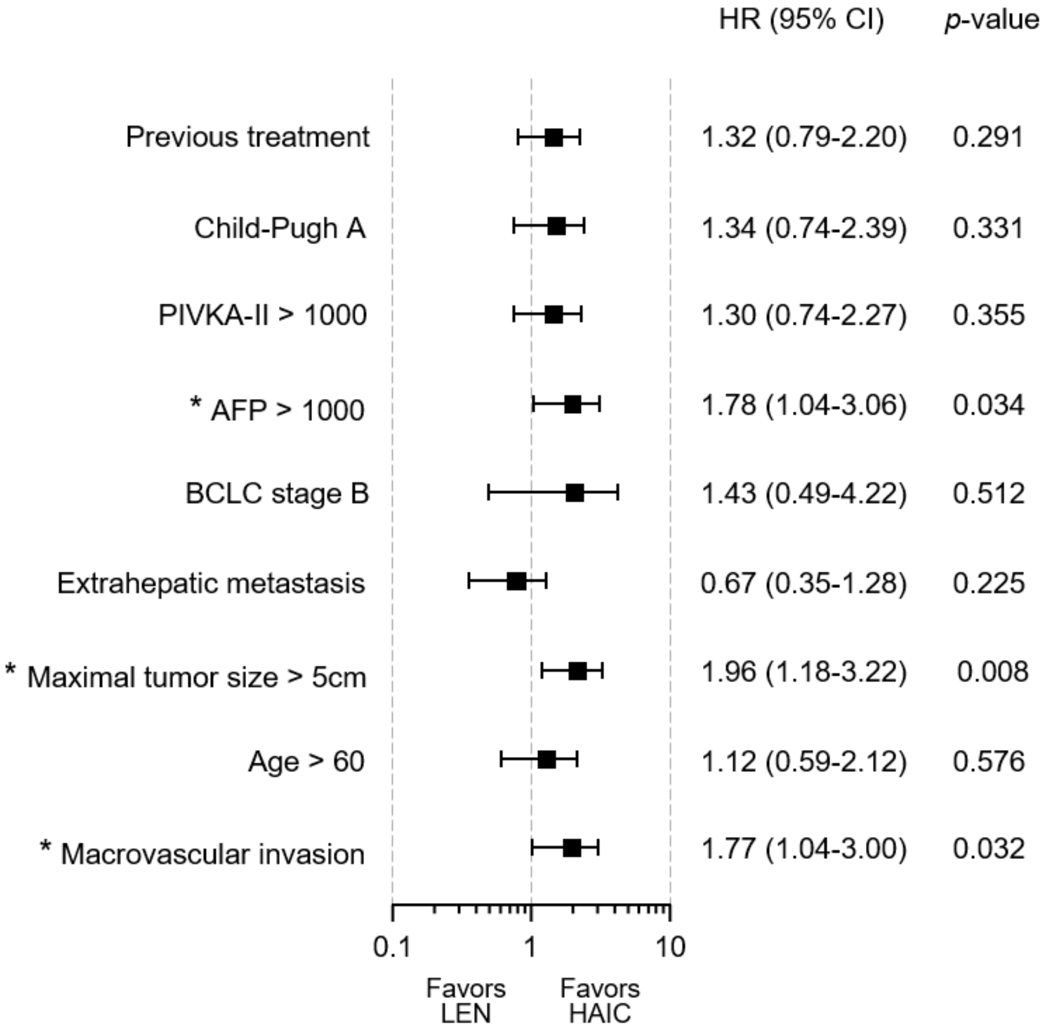

Figure S3. Forest plot comparing lenvatinib and HAIC group which representing hazard ratio, p-value, and median OS in each subgroup. *: statistically significant; CI: confidence interval; HR: hazard ratio.

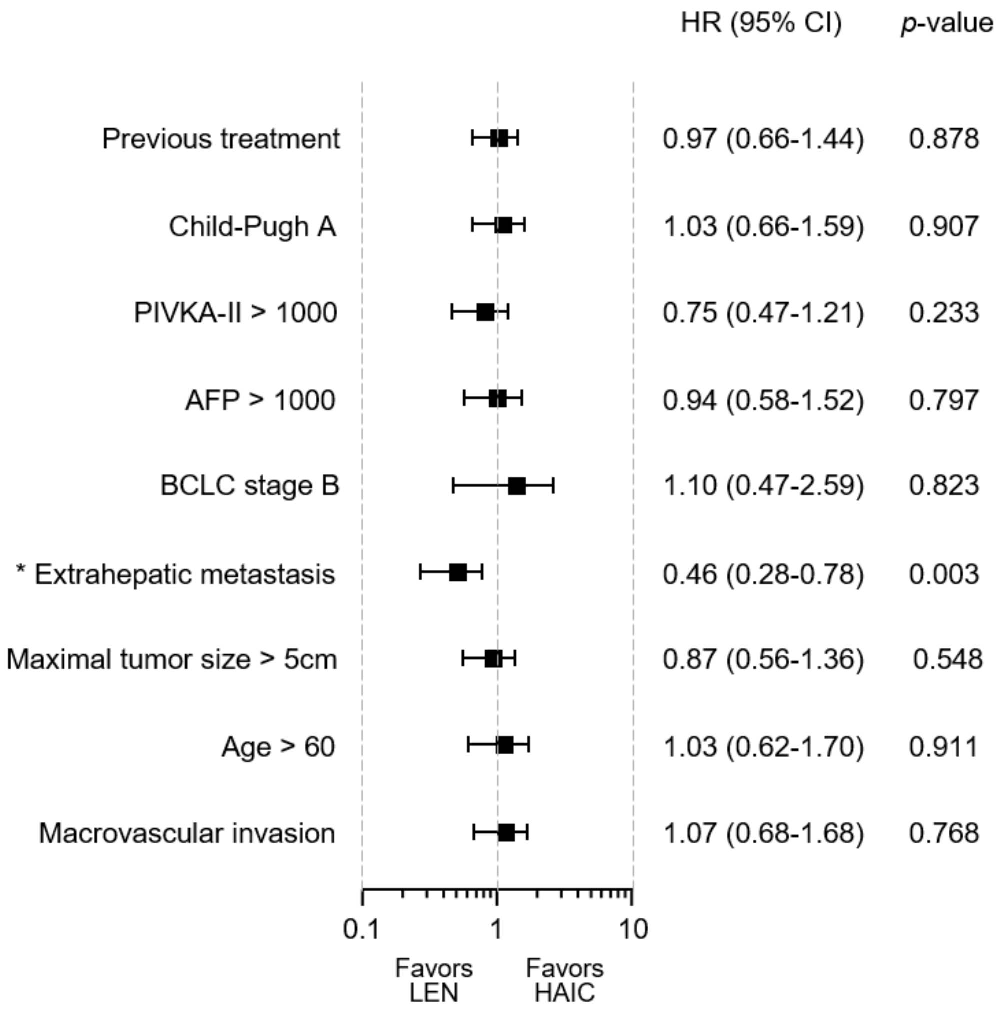

Figure S4. Forest plot comparing lenvatinib and HAIC group which representing hazard ratio, *p*-value, and median PFS in each subgroup. *: statistically significant.

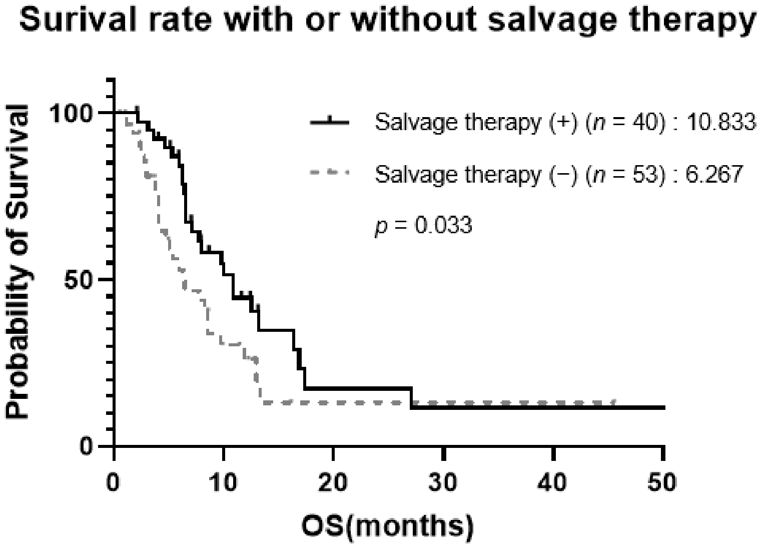

Figure S5. Kaplan-Meier curve representing survival difference between patients with salvage therapy and without salvage therapy at the end of HAIC or lenvatinib treatment, in PSM cohort.

Table S1. Previous treatment history before lenvatinib or HAIC treatment classified according to the BCLC stage.

	Lenvatinib			HAIC		
	**BCLC B (*n* = 14)**	**BCLC C (*n* = 57)**	**Total (*n* = 71)**	**BCLC B (*n* = 25)**	**BCLC C (*n* = 148)**	**Total (*n* = 173)**
TACE	10	38	48 (67.6)	17	63	80 (46.2)
TARE	0	1	1 (1.4)	0	4	4 (2.3)
RFA	3	3	6 (8.5)	4	10	14 (8.1)
HAIC	4	7	11 (15.5)	-	-	-
SBRT	1	15	16 (22.5)	0	13	13 (7.5)
Surgical resection	1	9	10 (14.1)	4	11	15 (8.7)
Systemic chemotherapy (overlapped)	0	6	6 (8.5)	2	10	12 (6.9)
Sorafenib	0	4	4 (5.6)	1	9	10 (5.8)
Lenvatinib	-	-	-	1	1	2 (1.2)
Regorafenib	0	2	2 (2.8)	0	3	3 (1.7)
Cabozantinib	0	1	1 (1.4)	0	0	0 (0)
Nivolumab	0	2	2 (2.8)	0	1	1 (0.6)

Data are given as *n* (%). HAIC, hepatic arterial infusion chemotherapy; TACE, transarterial chemoembolization; TARE, transarterial radioembolization; RFA, radiofrequency ablation; SBRT, Stereotactic body radiation therapy. BCLC: Barcelona Clinic Liver Cancer.

Table S2. Salvage therapy following lenvatinib or HAIC.

	**Lenvatinib (n = 46)**	**HAIC (n = 47)**	p
Salvage therapy	14 (30.4)	26 (55.3)	0.015
Sorafenib	1	11	
Lenvatinib	-	5	
Nivolumab	4	1	
Regorafenib	2	0	
Ramucirumab	0	1	
Atezolizumab-Bevacizumab	0	1	
TACE	3	6	
HAIC	1	-	
TARE	0	1	
Systemic cytotoxic chemotherapy	3	0	

Data are given as n (%). HAIC, hepatic arterial infusion chemotherapy; TACE, transarterial chemoembolization; TARE, transarterial radioembolization.
